# Driving south: a multi-gene phylogeny of the brown algal family Fucaceae reveals relationships and recent drivers of a marine radiation

**DOI:** 10.1186/1471-2148-11-371

**Published:** 2011-12-21

**Authors:** Fernando G Cánovas, Catarina F Mota, Ester A Serrão, Gareth A Pearson

**Affiliations:** 1 CCMAR, CIMAR-Laboratório Associado, Universidade do Algarve, Gambelas 8005-139, Faro, Portugal

## Abstract

**Background:**

Understanding the processes driving speciation in marine ecosystems remained a challenge until recently, due to the unclear nature of dispersal boundaries. However, recent evidence for marine adaptive radiations and ecological speciation, as well as previously undetected patterns of cryptic speciation is overturning this view. Here, we use multi-gene phylogenetics to infer the family-level evolutionary history of Fucaceae (intertidal brown algae of the northern Pacific and Atlantic) in order to investigate recent and unique patterns of radiative speciation in the genus *Fucus *in the Atlantic, in contrast with the mainly monospecific extant genera.

**Results:**

We developed a set of markers from 13 protein coding genes based on polymorphic cDNA from EST libraries, which provided novel resolution allowing estimation of ancestral character states and a detailed reconstruction of the recent radiative history. Phylogenetic reconstructions yielded similar topologies and revealed four independent trans-Arctic colonization events by Fucaceae lineages, two of which also involved transitions from hermaphroditism to dioecy associated with Atlantic invasions. More recently, reversion of dioecious ancestral lineages towards hermaphroditism has occurred in the genus *Fucus*, particularly coinciding with colonization of more extreme habitats. Novel lineages in the genus *Fucus *were also revealed in association with southern habitats. These most recent speciation events occurred during the Pleistocene glaciations and coincided with a shift towards selfing mating systems, generally southward shifts in distribution, and invasion of novel habitats.

**Conclusions:**

Diversification of the family occurred in the Late-Mid Miocene, with at least four independent trans-Artic lineage crossings coincident with two reproductive mode transitions. The genus *Fucus *arose in the Pliocene but radiated within a relatively short time frame about 2.5 million years ago. Current species distributions of *Fucus *suggest that climatic factors promoted differentiation between the two major clades, while the recent and rapid species radiation in the temperate clade during Pleistocene glacial cycles coincided with several potential speciation drivers.

## Background

Most of the world's biodiversity occurs in the oceans, but understanding the processes that drive speciation in marine ecosystems remains a challenge particularly due to the perceived scarcity of geographical barriers to gene flow [1]. Although much marine diversity stems from climate-driven vicariant and colonization events [2,3], the accumulation of phylogenetic information is revealing that a considerable amount of diversity arises during adaptive radiations [4,5], these periods of rapid speciation associated with diversification into multiple ecological niches (e.g., [6,7]), have also been shown to occur in marine systems where barriers to dispersal are not obvious [8].

Ecotypic divergence in response to strong environmental gradients or novel habitats (e.g., [8-11]) is a form of ecology-driven divergent selection that can cause population substructuring and differentiation [12]. Reproductive isolation occurs later, favouring assortative mating and facilitating speciation [13]. Mating system and reproductive ecology can also play an important role in marine speciation (e.g., [14]), although they remain under-studied in this environment. A major question is whether the evolution of hermaphroditic selfing entities from outcrossing lineages is a major trend in the sea, in common with terrestrial plant mating system evolution [15]. The evolutionary shift toward selfing increases colonization potential and reproductive assurance, while also serving to maintain local adaptations in stressful environments at the cost of genetic diversity and evolvability [16].

Geographical events driving speciation by vicariance or colonization have raised most interest in marine systems, and one of the most significant in the northern hemisphere was the opening of the Bering Strait. The formation of a marine connection between the North Pacific and the Arctic and North Atlantic Oceans [17,18] allowed trans-oceanic dispersal and divergence between Pacific and Atlantic sister taxa (e.g., [19]). Although the Pliocene opening of the Bering Strait has been placed at ca. 5.5 - 5.4 Ma [18], geomorphological and biological data [20] indicated that earlier openings possibly occurred in the Late Miocene. After the Pliocene opening, current patterns initially favoured Atlantic to Pacific exchanges [17] until ca. 3.5 Ma, after the closure of the Isthmus of Panama. The period of global cooling leading to the quaternary ice ages (starting ca. 1.8 Ma) began a series of oscillations in sea level and Arctic Ocean ice coverage, during which the Bering Strait closed and reopened at least six times [20]. Warmer periods coincided with higher trans-Arctic water flow, favouring inter-ocean dispersal events [21].

The brown algal family Fucaceae constitutes an important ecosystem-structuring component of cold to temperate intertidal communities in the North Pacific and North Atlantic Oceans. The wide northern hemisphere distribution of Fucaceae contrasts with their Australasian endemic sister families. This is thought to result from a trans-equatorial crossing with subsequent radiation in the northern hemisphere, a pattern paralleled in other families [22]. Ancestors of the Atlantic Fucaceae genera *Ascophyllum, Pelvetia *and *Fucus*, are hypothesized to have invaded the Atlantic through the Arctic during the last opening of the Bering Strait [23]. *Fucus *is the only Fucaceae genus that radiated extensively in the North Atlantic [23,24]. The cause of this process remains a challenging question that is only beginning to be understood [11,25,26]. Most extant genera within Fucaceae are, in strong contrast with *Fucus*, species-poor or monospecific. This allowed us to investigate which processes and events are associated with marine species radiations.

Speciation in *Fucus *may be associated with habitat-specificity (e.g., [11,25,27]) and variation in mating system and reproductive mode (e.g. [28-31]), with a biogeographic history shaped by glacial cycle-induced range shifts and secondary contact [26,32-36]. Although the phylogenetic history of the genus has never been fully reconstructed despite several attempts, two major clades were identified previously using nuclear [23] and mitochondrial DNA markers [24]. The first clade is northern, cold-water and relatively stress-susceptible (lineage 1 in [24]), and contains *F. serratus *and *F. distichus *(*sensu lato *[32]). The second clade (lineage 2) has a more southern extension with generally greater stress-tolerance, and contains *F. ceranoides, F. vesiculosus, F. spiralis, F. guiryi, F. virsoides *and *F. radicans*.

Our aim is to provide insight into marine speciation processes by inferring the phylogeny of the Fucaceae family. The study is particularly focused on unravelling the evolutionary history of radiative speciation within the genus *Fucus*, particularly the very speciose clade 2 (see below). In order to do this, we developed phylogenetic markers and used explicit biogeographic sampling of distinct populations and potentially novel species/entities suspected in clade 2 [11,25-27]. We also provide a temporal evolutionary hypothesis by calibrating the obtained phylogenies in geological time using the fossil record from extinct members of brown algae [37] and information from a dated brown algal multilocus phylogeny [38]. The phylogenetic framework is integrated with paleo-reconstructions from the Earth's climatic history [39-41] and landmass trends from plate tectonic movements [42], to provide a hypothesis explaining the major historical events in the evolutionary history of Fucaceae.

## Results

### Sequences and trees

The dataset for the multi-gene phylogenetic analysis comprised 4878 aligned bp (1626 amino acids) stored in 13 partitions, each representing a different protein coding region (Additional file 1), based on the cDNA synthesized from isolated RNA from 84 individuals representing all genera in the Fucaceae.

The analyses yielded a well-defined phylogenetic hypothesis for the Fucaceae. Replicate runs of the Bayesian approach (see methods) converged onto similar posterior distributions after less than 5% of the 10^6 ^generations. Phylogenetic reconstructions provided high confidence except for the branching of taxa *F. radicans *and *F. gardneri*. Relationships among *Fucus *species, with the exception of the most recently diverged entity *F. radicans *were resolved using this cDNA dataset.

### Genomic divergence at protein-coding loci

Analysis of the 13 partial coding sequences provided 395 variable sites, 31 of which were identified as singletons (see Additional file 2 for accession numbers). Intra-specific nucleotide (nt) variability in the genomic data set was low, ranging from zero to 10 single nucleotide polymorphisms (SNP) in the most diverse species, *Pelvetiopsis limitata *(Table 1). Intra-specific diversity, measured as the average number of nucleotide differences between pairwise sequences across all loci (Table 1), revealed two significantly more diverse species: *P. limitata *(14.67) and *Hesperophycus californicus *(7.33). All the other species showed average values below 3 nucleotide differences.

**Table 1:** Genomic data estimations

		Fucus clade 1			Fucus clade 2											
	Species	*F.serratus*	*F.evanescens*	*F.gardneri*	*F.radicans*	*F.ceranoides*	*F.spiralis*	*F.guiryi*	*F.virsoides*	*F. vesiculosus Northern*	*F. vesiculosus Southern*	H.ca*lifornicus*	P.ca*naliculata*	*P.limitata*	S.com*pressa*	*A.nodosum*
	*F. serratus*	2.56	0.004 ± 0.001	0.004 ± 0.001	0.006 ± 0.001	0.007 ± 0.002	0.006 ± 0.001	0.007 ± 0.001	0.008 ± 0.002	0.006 ± 0.001	0.006 ± 0.001	0.014 ± 0.003	0.026 ± 0.004	0.014 ± 0.003	0.027 ± 0.005	0.026 ± 0.004
Fucus lineage 1	*F. evanescens*	**0.35**	2.67	0.001 ± 0.000	0.007 ± 0.002	0.008 ± 0.002	0.007 ± 0.002	0.007 ± 0.002	0.009 ± 0.002	0.007 ± 0.002	0.007 ± 0.002	0.015 ± 0.003	0.027 ± 0.005	0.015 ± 0.003	0.028 ± 0.005	0.027 ± 0.005
	*F. gardneri*	**0.38**	**0.09**	0.00	0.007 ± 0.002	0.008 ± 0.002	0.007 ± 0.002	0.008 ± 0.002	0.009 ± 0.002	0.008 ± 0.002	0.008 ± 0.002	0.016 ± 0.003	0.027 ± 0.005	0.015 ± 0.003	0.029 ± 0.005	0.027 ± 0.005
	*F. radicans*	**0.61**	**0.68**	**0.72**	0.00	0.002 ± 0.001	**0.000 ± 0.000**	**0.000 ± 0.000**	0.002 ± 0.001	**0.000 ± 0.000**	**0.000 ± 0.000**	0.014 ± 0.003	0.024 ± 0.004	0.013 ± 0.003	0.025 ± 0.005	0.025 ± 0.004
	*F. ceranoides*	**0.67**	**0.75**	**0.79**	**0.22**	2.67	0.002 ± 0.001	0.003 ± 0.001	0.004 ± 0.001	0.002 ± 0.001	0.002 ± 0.001	0.014 ± 0.003	0.025 ± 0.004	0.013 ± 0.003	0.026 ± 0.004	0.025 ± 0.004
Fucus lineage 2	*F. spiralis*	**0.61**	**0.69**	**0.72**	**0**	**0.22**	0.44	**0.000 ± 0.000**	0.002 ± 0.001	**0.000 ± 0.000**	**0.000 ± 0.000**	0.014 ± 0.003	0.024 ± 0.004	0.013 ± 0.003	0.025 ± 0.005	0.025 ± 0.004
	*F. guiryi*	**0.64**	**0.71**	**0.75**	**0.03**	**0.25**	**0.04**	1.27	0.002 ± 0.001	**0.000 ± 0.000**	**0.000 ± 0.000**	0.014 ± 0.003	0.025 ± 0.004	0.013 ± 0.003	0.026 ± 0.005	0.025 ± 0.004
	*F. virsoides*	**0.78**	**0.86**	**0.9**	**0.18**	**0.4**	**0.18**	**0.21**	2.00	0.002 ± 0.001	0.002 ± 0.001	0.015 ± 0.003	0.026 ± 0.004	0.015 ± 0.003	0.027 ± 0.005	0.027 ± 0.005
	*F. vesiculosus Northern*	**0.62**	**0.69**	**0.73**	**0.01**	**0.23**	**0.02**	**0.04**	**0.19**	0.78	**0.000 ± 0.000**	0.014 ± 0.003	0.024 ± 0.004	0.013 ± 0.003	0.026 ± 0.004	0.025 ± 0.004
	*F. vesiculosus Southern*	**0.61**	**0.69**	**0.73**	**0.01**	**0.23**	**0.01**	**0.04**	**0.19**	**0.02**	0.78	0.014 ± 0.004	0.024 ± 0.005	0.013 ± 0.004	0.025 ± 0.005	0.025 ± 0.005
	*H. californicus*	1.38	1.46	1.49	1.31	1.33	1.31	1.34	1.48	1.32	1.31	7.33	0.025 ± 0.004	0.008 ± 0.002	0.027 ± 0.005	0.025 ± 0.004
	*P. canaliculata*	2.47	2.55	2.58	2.32	2.34	2.32	2.35	2.49	2.33	2.32	2.41	0.00	0.024 ± 0.004	0.027 ± 0.005	0.025 ± 0.004
	*P. limitata*	1.34	1.41	1.45	1.22	1.25	1.23	1.25	1.40	1.23	1.23	**0.74**	2.25	14.67	0.024 ± 0.004	0.023 ± 0.004
	*S. compressa*	2.57	2.69	2.72	2.41	2.44	2.42	2.44	2.59	2.42	2.42	2.60	2.54	2.30	0.67	0.014 ± 0.003

Number of base substitutions per site and standard error (1000 bootstraps) calculated using the maximum composite likelihood method ([92] provided in MEGA v4.1 [93]; above diagonal) and percentage of divergence based on average number of differences (below diagonal) between species. Divergence levels lower than 1% as well as zero substitutions are emphasized. Diagonal elements: number of pairwise differences within species.

The highest inter-species differentiation was seen between the genus *Fucus *and all the other Fucaceae, ranging from 1.58% to a maximum of 3.05% (0.013 to 0.027 average number of substitutions per site). The lowest differentiation between genera was that between *H. californicus *and *P. limitata *(0.87% and 0.008 ± 0.002 average number of substitutions per site), which is close to values found between species of the two major *Fucus *clades (less than 0.8% and 0.008 ± 0.002). The heat shock 90 family protein-coding gene had a 3-codon insertion in *Silvetia compressa *that clearly differentiated this species, despite it displaying low genetic differentiation from a sister genus, here *A. nodosum *(1.5%).

### Multi-gene phylogeny of the family Fucaceae

**Figure 1:**
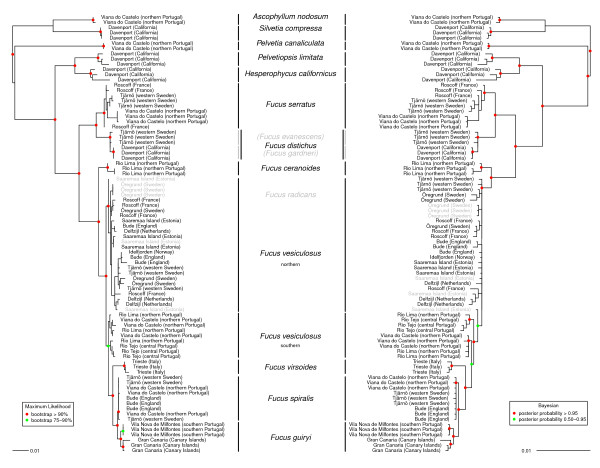
**Multi-gene phylogenetic reconstruction from 13 cDNA loci**. Multi-gene phylogenetic reconstructions using 13 nuclear transcriptomic regions. Shown are the 50% majority rule consensus tree of maximum likelihood bootstraps (left) and the 50% majority rule percentage of support for clades given by Bayesian posterior probabilities from one million generation MCMC analysis (right). cDNA trees were rooted using as outgroup the most basal genera, *Ascophyllum *and *Silvetia*, determined as basal according to phylogenetic re-analysis of Fucaceae ITS data using its sister families as outgroup [23] (see details in Methods section for the analyses performed based on [23] and Additional file 2 for the corresponding phylogenetic reconstruction).

Both maximum likelihood and Bayesian-based reconstruction algorithms yielded similar topologies (Figure 1), differing mostly in the branch lengths and support values. All current species were resolved except the recent Baltic species *F. radicans*, and all nodes that split different species showed high support for both algorithms. Phylogenies built using cDNA nucleotide sequences therefore resulted in much improved resolution over previously used markers, despite lower genetic distances than earlier described with ITS. Re-analysis of ITS data [23] confidently inferred the *Ascophyllum - Silvetia *clade to root the remaining divergence events in the Fucaceae using the 13 cDNA loci.

The 13 protein-coding genes identified the same two major clades within *Fucus *as ITS ([23] and reanalyzed data in Additional file 3) and mitochondrial DNA [24]. In *Fucus *clade 1, two subclades were again recovered. Dioecious *F. serratus *and the hermaphroditic group corresponding to *F. distichus sensu lato*, in which our sampling of the geographic extremes revealed low intra-specific divergence.

**Figure 2:**
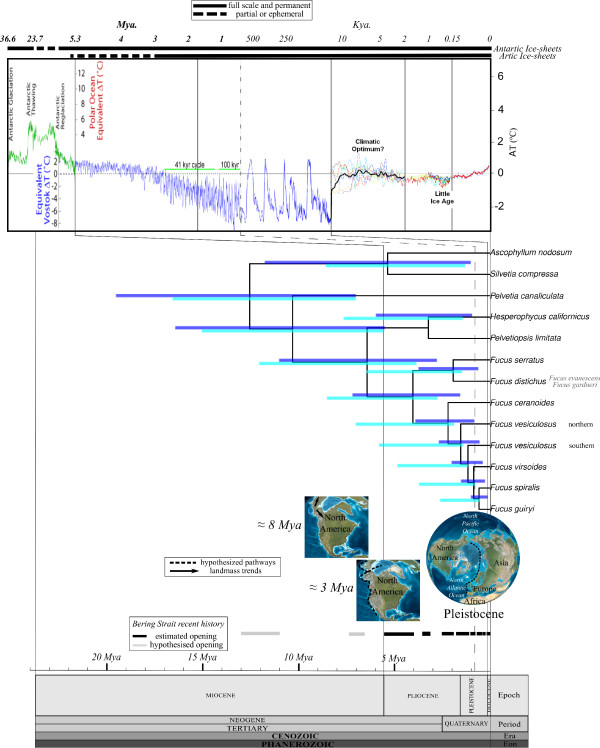
**Bayesian dating of Fucaceae diversification**. Simplified Bayesian dated phylogenetic reconstruction using the 13 coding loci. Node ages in million years (Myr) with their 95% HPD interval for both expansion growth (violet bars) and Yule speciation (cyan bars) models correspond to the time scale at the bottom of the Figure. Polytomies within species were collapsed for clarity, extracting the most divergent individuals (= leaf) from the Bayesian dating of Fucaceae diversification (for full tree see Additional file 4) Each paleogeographic reconstruction is placed at the estimated age (reproduced with permission of Dr. R. Blakey). Temperature graph shows paleoclimate reconstructions according to Zachos et al. ([40]; Paleocene to Miocene), Lisiecki et al. ([41]; Pliocene to Pleistocene) and Petit et al. ([39], Holocene) (reproduced with permission of Dr. R.A. Rohde). The ages and their correlation to the names on the geological timescale are based on Gradstein et al. [91]. Recent history of the Bering Strait is shown with the estimated and hypothesized openings [18,20].

In contrast with the polytomy found previously [23,24], species relationships within clade 2 were resolved (Figure 1), with the exception of *F. radicans *(see below). The earliest diverging lineage leads to the estuarine species *F. ceranoides*. This is followed by the discovery that *F. vesiculosus *is not monophyletic, but is split according to geographical location of the samples into a northern (splitting earlier from the remaining species; Figure 1, ML phylogeny), and a southern clade. The latter shares a common ancestor with the hermaphroditic species in this lineage. The southern *F. vesiculosus *samples appear to form two distinct clades of geographically similar individuals (Figure 1, Bayesian phylogeny) but they are grouped in a single clade in the Bayesian inferences based on the coalescent and Yule speciation models (Figure 2 and Additional file 4). The recently derived species *F. radicans *was not resolved and grouped with sympatric northern *F. vesiculosus*. All of these dioecious species/entities were basal to the clade containing the three hermaphroditic species, the Mediterranean endemic *F. virsoides *branching first, followed by the clade containing *F. spiralis *and the recently described southern species *F. guiryi *[11], that was clearly differentiated from *F. spiralis *with high node values for both algorithms. Phylogenetic trees in Figure 1 were built after excluding *F. guiryi *individuals from the introgressed contact range (see discussion). The resulting trees including those individuals (shown in Additional file 5) show the effects of introgressed individuals in confounding the inference of vertical lineage splitting [11].

### Evolutionary rates and molecular dating

Bayesian MCMC inference resulted in an estimate of the mean evolutionary rate across Fucaceae of 0.0016 substitutions per thousand years (95% confidence interval 0.0008 to 0.0025). We emphasize that, taking into account the confidence intervals, the evolutionary rates for the separate coding genes largely overlapped and the coefficient of variation across the tree was 0.6. The nucleotide substitutions per site range from values close to zero for comparisons within *Fucus *spp. up to 0.029 ± 0.005 for the whole family (*S. compressa *and *F. gardneri*; Table 1).

Coalescent theory and the Yule speciation model were used to evaluate the history of Fucaceae (Figure 2). Trees agree well with Bayesian and ML phylogenetic reconstructions (Figure 1). Both demographic models broadly coincided when used to infer dates for the nodes placed near the origin of all Fucaceae genera, but differed considerably in dating recent speciation events, particularly within *Fucus*. The time intervals reported are maximally conservative and correspond to the range for both demographic models together. Our molecular dating leads to an estimate for the origin of the diversification of Fucaceae around 19.5-7.0 million years ago (Ma) (Figure 2 and Additional file 6). The origin of the lineage leading to *Pelvetia canaliculata*, eventually resulting in an Atlantic invasion, was dated at 16.4 to 5.4 Ma. Divergence between the lineages leading to *Ascophyllum nodosum *and to the genus *Silvetia *(11.7 to 1 Ma) was coincident in time with the split of the lineage leading to *H. californicus *and *P. limitata *from the *Fucus *genus lineage (12.2 to 2.7 Ma). Both of these splits correspond to a Pacific-Atlantic crossing by members of the lineages now represented by the genera *Ascophyllum *and *Fucus *in the Atlantic. The diversification of the genus *Fucus *into two clades was estimated at 9.5 to 1.6 Ma. All the predicted speciation events within each *Fucus *clade were placed within the last 3.8 million years (Myr).

### Tests of mating system evolutionary hypotheses

**Figure 3:**
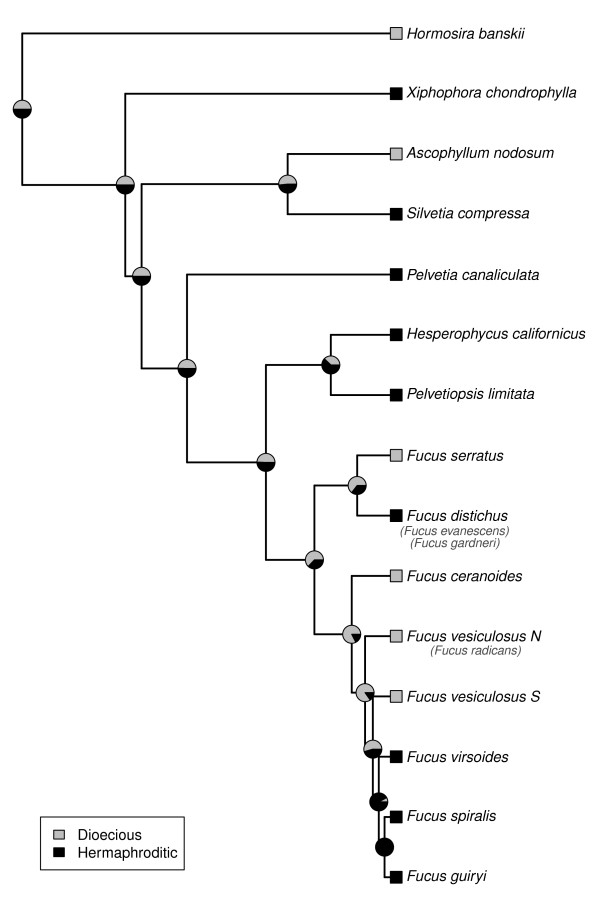
**Mapping character evolution**. Mapping character evolution for mating system (dioecious *vs*. hermaphroditic) on the simplified Bayesian dated phylogenetic reconstruction (see Figure 2 and methods section), where scaled likelihood of each character state is represented as pie graphics on the nodes [80].

The best fitting model for the different diversification hypotheses related to the evolution of mating systems (see Table 2 and estimation of ancestral character states and diversification in the Methods section) was the one-parameter Markov k-state model (MK1; *AIC *= 104.522). This model indicates that speciation (λ) and extinction (μ) rates were state-independent λ*_dioecious _*= λ*_hermaphroditic _*= 0.24; μ*_dioecious _*= λ*_hermaphroditic _*= 0.14) and that the transition between character states was also bidirectional and state-independent (*q01 *= *q10 *= 0.56). Ancestral state reconstruction from the scaled likelihood of every state of the character (Figure 3) resulted in poor resolution of deeper nodes, showing equal likelihood for either character state (dioecious *vs*. hermaphroditic). The node describing the state of the mrca of all *Fucus *species showed higher scaled likelihood for the state of dioecy (*logLik_scaled _*= 0.63), as well as the other nodes involved in the evolution of the genus (*logLik_scaled _*ranged from 0.54 to 0.84). However the two more recent ancestors of *F. virsoides, F. spiralis *and *F. guiryi *were estimated to have been hermaphroditic (*logLik_scaled _*= 0.92 and 0.99, respectively). The common ancestor of *Hesperophycus *and *Pelvetiopsis *was also estimated as having been hermaphroditic (*logLik_scaled _*= 0.62).

**Table 2:** Tests of mating system evolution hypotheses

*Scenario*	*Df*	*λ0*	*λ1*	*μ0*	*μ1*	*q01*	*q10*	*logLik*	*AIC*	*P*
**Asymmetric parameters**	6	0.000	0.289	0.398	0.000	0.441	0.524	-48.299	108.597	
**Asymmetric speciation**	4	0.000	0.350	*μ1~μ0*		*q01~q10*		-48.519	105.040	·
**Asymmetric extinction**	4	*λ1~λ0*		0.364	0.000	*q01~q10*		-48.997	106.000	·
**Source (dioecious)-sink system**	4	*λ1~λ0*		*μ1~μ0*		0.086	-	-54.358	114.720	*
**Source (hermaphoditic)-sink system**	4	*λ1~λ0*		*μ1~μ0*		-	0.073	-52.686	111.370	**
**Symmetric parameters (MK1)**	3	*λ1~λ0*		*μ1~μ0*		*q01~q10*		-49.261	104.522	·
**Sink-sink system (MK2)**	4	*λ1~λ0*		*μ1~μ0*		0.758	0.538	-49.068	106.135	·
Significance codes: 0 < *** < 0.001 < ** < 0.01 < * < 0.05 < · < 0.1 < NS < 1										

Speciation (λ), extinction (μ) and transition (*q*) rates between the two states of the mating system character (0, dioecious; 1, hermaphroditic). Complete asymmetric model and different scenarios of state-independent and state-dependent diversification (rates constrained to be equal), and unidirectional and bidirectional transitions were tested: 1) asymmetric model; 2) asymmetric speciation or state-independent extinction and transition rates; 3) asymmetric extinction or state-independent speciation and transition rates; 4) Source (dioecious)- sink system or state-independent diversification rates and transition from hermaphroditic to dioecious state constrained to 0; 5) Source (hermaphroditic)- sink system or state-independent diversification rates and transition from dioecious to hermaphroditic state constrained to 0; 6) symmetric parameters or MK1 model; 7) Sink-sink system or MK2 model [81-84,86]. Log-likelihood, Akaike information criteria (AIC) and log-likelihood ratio test (P) are also provided for comparison between models.

### Tests of geographic hypotheses related to the Bering Strait oceanic boundary

**Figure 4:**
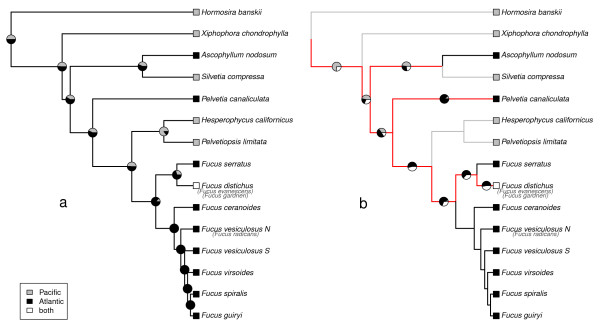
**Mapping character evolution**. Mapping character evolution for biogeographic range, where scaled likelihood of each character state is represented as pie graphics on the nodes (a) [80]. Ancestral range subdivision/inheritance biogeographic scenarios, where color on branches shows hypothesised scenario (b) [88,89]; unsolved branch-scenarios are coloured in red but then, scaled likelihood meaning either side of Bering Strait, thus Arctic and Atlantic *vs*. Pacific is provided. Character evolution is traced on the simplified Bayesian dated phylogenetic reconstruction (see Figure 2 and methods section). Note that *F. distichus *was analysed as either Atlantic and Pacific.

The testing of hypotheses related to geographic (i.e., Pacific *vs*. Atlantic/Arctic) origin and diversification (see Table 3 and estimation of ancestral character states and diversification from the Methods section) showed the GeoSSE model as having the best fit (*AIC *= 107.66, *log-likelihood *= -46.83). The model indicates that diversification rates were state-dependent (*s_Pacific _*= 0.195; *s_Atlantic _*= 0.019; *x_Pacific _*= 0.049; *x_Atlantic _*= 0.000), and that the dispersal rates between the two geographic regions (both sides of the Bering Strait) were almost unidirectional from the Pacific (*d_Pacific to Atlantic _*= 0.075). The DEC model also reported low dispersal (0.032) and extinction (0.000) rates (*log.likelihood *= -13.25). Ancestral state reconstruction was also performed for the sink-sink GeoSSE model (second best model like MK2) and DEC models (Figure 4a and 4b, respectively). Deeper nodes were poorly resolved by the sink-sink model, showing similar scaled likelihoods for either character state (Pacific *vs*. Atlantic; Figure 4a), but DEC provided better estimates for the alternative scenarios (branches in Figure 4b). Estimates of the geographic origin of the family were similar for Pacific and Atlantic Oceans using the GeoSSE model (*logLik_scaled _*= 0.42 and 0.58, respectively), while the DEC model placed the origin in the Pacific (*logLik_scaled _*= 0.47) or in both biogeographic areas across the Bering Strait (*logLik_scaled _*= 0.32; pie on nodes in Figure 4b). This last observation agrees with the rate of between-region mode of speciation obtained by the GeoSSE model (*s_Pacific-Atlantic _*= 1.225) that was higher than within-region speciation rates. Alternatively, these results also agree with hypothetical divergence along the boundary between both regions in the Arctic Ocean. The most recent common ancestor to *Fucus *was estimated as Atlantic (*logLik_scaled _*= 0.86 and 0.63 for both GeoSSE and DEC models, respectively). Finally, both models predicted an Atlantic ancestor of *F. serratus *and *F. distichus *(clade 1; *logLik_scaled _*= 0.70 for the nodes and 0.53 for the inheritance scenario).

**Table 3:** Tests of biogeographical hypotheses

*Scenario*	*Df*	*sA*	*sB*	*sAB*	*xA*	*xB*	*dA*	*dB*	*logLik*	*AIC*	*P*
**Assymetric parameters**	7	0.195	0.019	1.225	0.049	0.000	0.070	0.000	-46.828	107.656	
**Assymetric speciation**	5	0.164	0.020	1.225	*xA~xB*		*dA~dB*		-48.775	107.550	NS
**Assymetric extinction**	5	*sA~sB*		1.225	0.058	0.029	*dA~dB*		-51.580	113.160	**
**Symmetric parameters**	4	*sA~sB*		1.225	*xA~xB*		*dA~dB*		-51.636	111.270	**
**Sink-sink system**	5	*sA~sB*		1.225	*xA~xB*		0.075	0.000	-50.172	110.340	*
**Unconstrained DEC**	2	-		-	0.032		0.000		-13.25	-	-
**Stratified biogeographical DEC model**	2	-		-	0.010		0.061		-16.45	-	-
Significance codes: 0 < *** < 0.001 < ** < 0.01 < * < 0.05 < · < 0.1 < NS < 1											

Speciation (*s*), extinction (*x*) and dispersal (*d*) rates between the two biogeographical regions on either side of the Bering Strait (A, Pacific Ocean; B, Atlantic Ocean, including most of the Arctic). Complete asymmetric model and different scenarios of state-independent and state-dependent diversification (rates constrained to be equal), and unidirectional and bidirectional dispersal were tested: 1) asymmetric model; 2) asymmetric speciation, state-independent extinction and dispersal rates; 3) asymmetric extinction, state-independent speciation and dispersal rates; 4) symmetric parameters; 7) Sink-sink system [86]. Log-likelihood, Akaike information criteria (AIC) and log-likelihood ratio test (P) are also provided for comparison between models. Dispersal and local extinction parameters estimated by DEC models are also shown [88,89].

## Discussion

A multi-gene phylogenetic approach resulted in a much finer resolution of relationships at the tips of the tree compared with previous phylogenies. This framework allowed the estimation of dates of divergence and patterns of speciation across the family and within the recently radiated genus *Fucus*.

### Dating inter-ocean divergence events in Fucaceae

The models used returned similar dated intervals on deeper nodes corresponding to the splitting events of ancestral Fucaceae lineages, but were less congruent in dating recent speciation events. This is likely due to the constraints of the priors used [43,44]. Although we remain conservative by reporting the range for both models, the narrower and more recent coalescent-based intervals at the tips of the tree are more in agreement with the biological processes associated with speciation in these taxa [45].

The most likely origin of the Fucaceae is in the Pacific Ocean during the mid to late Miocene (19.5-7.0 Ma, estimated based on 23-7 Ma from [37] and 19.4-8.0 Ma from ITS; see Additional file 6), when an ancestor of the Fucaceae might have been able to colonize the North Pacific, splitting from the Australasian sister lineages Xiphophoraceae and Hormosiraceae [23,38]. Despite support for both alternative hypotheses for the Fucaceae geographic origin, a Pacific origin involves a more direct route from the southern (Australasia) to the northern Pacific (and is supported by diversification rates and the DEC model), whereas the alternative hypothesis of an Atlantic origin requires a more complex dispersal path. A Pacific origin is also consistent with the northward drift of the Australasian landmass towards Eurasia in the Miocene and a gradual decrease in global temperatures (14-12 Ma, see Figure 2; [40]), which would have favored crossing of the equatorial fringe. The origin of the Fucaceae would then be due to subsequent divergence in the North Pacific.

Our data indicate that four independent Fucaceae lineages crossed the Bering Strait. The first crossing, estimated at 16.4-5.4 Ma (Figure 2), involved the splitting of the Atlantic lineage leading to *Pelvetia canaliculata*, and could only have taken place during the earliest openings of the Bering Strait suggested for the Late Miocene (13.0-11.0 and 7.3-6.6 Ma; [20]). Pacific diatoms found in Atlantic marine sediments indicate the existence of a strait at that time [20], supporting such early Pacific-Atlantic colonizations. The alternatives to this scenario, other than methodological bias in dating, require either accelerated lineage divergence following the trans-Arctic crossing, or the start of divergence before the trans-Arctic crossing. The latter is unlikely because *Pelvetia *is currently monotypic with no extant Pacific representatives. While the extreme upper intertidal distribution and stress tolerance of Atlantic *P. canaliculata*, makes accelerated selective ecological divergence a plausible explanation, it is unnecessary to invoke it if earlier openings of the Bering Strait occurred [20]. A second (and probably later; 11.5-1.1 Ma, Figure 2) trans-Arctic crossing led to the Atlantic genus *Ascophyllum*, following a split from its Pacific sister genus *Silvetia*, coincident with the Bering Strait opening at 5.5-5.4 Ma [18]. These results contradict the previous ITS phylogeny of [23] but agree with these data after their re-analysis with better fit models (see methods). It revealed *Ascophyllum *as sister to the Pacific genus *Silvetia *and placed the *Ascophyllum-Silvetia *in a basal clade to the Fucaceae, a hypothesis also raised by [23].

The third (possibly simultaneous) trans-Arctic crossing, and the most successful in terms of subsequent speciation, was the split between the current *Hesperophycus-Pelvetiopsis *in the Pacific and the lineage leading to *Fucus*, of which all current species are Atlantic except the circum-Arctic *F. distichus *complex. This divergence, estimated at 12.2-2.7 Ma, coincides both in time and reproductive mode (shifting from hermaphroditic to dioecious) with the *Ascophyllum *lineage split from the *Silvetia *clade. The timing of both lineage splitting events leading to *Ascophyllum *and *Fucus *centers around the opening of the Bering Strait 5.5-5.4 Ma when, despite moving against the predominant Atlantic-Pacific flow, the warmer climate (see Figure 2) might have facilitated stepping stone colonization and migration across the Arctic. Ancestral state reconstructions (Figure 4) place the most recent common ancestor of *Fucus *in the Atlantic/Arctic ocean basin, suggesting that it was here that subsequent diversification took place. The alternative hypothesis, deserving further study, is that the opening of the Bering Strait led to a vicariant split between clade 1 in the Pacific and clade 2 in the Atlantic. An additional interesting question remains as to why, following similar colonization conditions by ecologically similar lineages, *Ascophyllum *is currently a monotypic genus whereas *Fucus *underwent relatively extensive speciation.

The fourth trans-Arctic crossing involved the evolutionary history of Arctic vicariance in *Fucus *clade 1. The ancestor to clade 1 was estimated as Atlantic (Figure 4), and the Atlantic-Pacific dichotomy might be more accurately described as Arctic to agree with the geographical and ecological range of current representatives. The ancestral state reconstruction implies that *F. serratus/F. distichus *diverged in the Atlantic and/or within the Arctic basin, which represent the same side of the Bering Strait, with subsequent invasion of the Pacific by the *F. distichus *lineage. Although Atlantic (previously named *F. evanescens*) and Pacific (previously named *F. gardneri*) samples of *F. distichus *used in this phylogeny correspond to the geographical extremes of the ranges found within the *F. distichus *complex [24,46], estimated Pacific-Atlantic divergence times based on coalescence are very recent (mid-Pleistocene) (Figure 2). Thus our data do not contradict the current designation of these lineages as a single species, *F. distichus *(see [32]), but do not rule out low levels of vicariant divergence (Figure 1), also in agreement with Coyer et al. [32].

### Driving south: a biogeographical hypothesis for the evolution of Fucus clade 2

The earliest branching member of the clade is the dioecious lineage *F. ceranoides*. The contemporary cold-temperate distribution of *F. ceranoides *from Norway to North Portugal is similar to the present day range of *F. serratus *in clade 1 [33], which has a coincident speciation time (Figure 2). Nuclear and organelle phylogenies for *F. ceranoides *are congruent in the southern part of the range, while to the north of the English Channel populations harbour exclusively introgressed organellar genomes captured from *F. vesiculosus *that have spread by genetic surfing during postglacial range expansion [36]. This is not the only case of organellar introgression in this clade [26], emphasizing that organellar sequences can be equivocal for phylogenetic inferences in taxa prone to introgression. *F. vesiculosus *was shown here to be polyphyletic. Two clades were well separated within *F. vesiculosus *according to their range distributions from: i) Iberia to the south *versus*, ii) the English Channel to the north. These are also differentiated at microsatellite loci [25,29,47], both in allelic frequencies and in the presence of private alleles, but were not recovered previously with mitochondrial markers [24,26], possibly due to masking by extensive organellar introgression-expansion dynamics that can take place in *Fucus *species [36]. Importantly, the southern *F. vesiculosus *share a common ancestor with the remaining members of the same lineage, all of which are hermaphroditic. The two divergent lineages in what is currently named *F. vesiculosus *coincide in present distribution with two marine glacial refugia (Iberia and Brittany; [3]). A split of southern *F. vesiculosus *into two clades suggested by certain analyses (Figure 1 and Additional file 4 and 5) deserves further investigation, but could result from introgressive signatures with *F. guiryi*, which may be found in sympatry in some regions [11,25,47], but not in the southernmost sites where the two species are allopatric [11,30] (Figure 1 and Additional file 5).

Divergence of the hermaphroditic lineage in clade 2 (leading to *F. virsoides, F. spiralis *and *F. guiryi*) from their dioecious sister lineage may have been driven or at least maintained by reproductive isolation derived from a selfing reproductive mode. Once a hermaphroditic lineage arises, selfing may follow rapidly, reinforcing genetic isolation and favouring subsequent differentiation [48]. Selfing can be advantageous in marginal and/or stressful habitats to conserve local adaptation and for reproductive assurance, both key selective pressures for intertidal broadcast spawners such as *Fucus *[49].

The earliest divergence within the hermaphroditic clade is *F. virsoides*, currently restricted to the northern Adriatic Sea, a possible remnant from a more extensive distribution during a cooler glacial period. More recently, the lineage split between *F. guiryi *and *F. spiralis *coincides with southern *vs*. northern ranges. Along the southern range, *Fucus *species are segregated by habitat, i.e., open coast (*F. guiryi*) *versus *estuaries and coastal lagoons (southern *F. vesiculosus*), whereas further north, where they co-occur, *F. guiryi *undergoes introgression [11,25,26,47], which was hypothesized to reflect the absence of reinforcement during allopatric evolution [47]. The phylogenetic position of the high intertidal *F. spiralis *reported here is incongruent with mitochondrial data [26], possibly another case of extensive organellar introgression in this genus.

Our data, like previous ITS and mitochondrial data [23,24], do not resolve the relationship between the recently described *F. radicans *and *F. vesiculosus*. This is unsurprising given the suggested timescale of divergence (hundreds to at most thousands of years [27]), since the opening of the Baltic Sea (ca. 7 Kya), possibly facilitated by high adaptive potential of the common ancestor with *F. vesiculosus *[10,50].

### Mating system evolution

The evolution of reproductive mode in the Fucaceae has followed a reticulate pattern of alternating dioecious and hermaphroditic lineages that challenges current understanding of mating system evolutionary trends (Figure 3; e.g., [15,16]). Methods to estimate the influence of species' traits on lineage diversification establish hermaphroditic lineages as ancestral in the family, evolving into dioecious lineages, folowed by switches from dioecy to hermaphroditism in the genus *Fucus*, contradicting earlier suggestions [24,51]. There is considerable support for hermaphroditism (cosexuality) as the ancestral state in plants [15], and simple genetic mechanisms of dioecious sex determination and sex chromosome evolution have been proposed (reviewed by [52,53]). It is intriguing that two of the three novel Atlantic lineages presumably coincided with a switch to dioecy (*Ascophyllum *and *Fucus*). The evolution of dioecy and increased evolutionary potential [16] may therefore have facilitated long-term establishment in the Atlantic, driven in part by the availability of extensive and novel habitats favouring large and dense populations. In contrast, hermaphroditic lineages are better colonizers of marginal habitats via increased reproductive assurance and the maintenance of locally adaptive traits.

The recent evolutionary trajectory of reproductive mode has been a switch towards hermaphroditism, and highly selfing mating systems, at least within *Fucus *lineage 2 [29,30]. The transition from outcrossing to selfing is common in plants [54], but with little evidence for reversion, suggesting an evolutionary dead-end [16,55]. This in turn suggests that the hermaphroditic ancestors of the dioecious lineages leading to *Ascophyllum *and *Fucus *were not highly selfing.

## Conclusions

The analysis of concatenated cDNA sequences from 13 partial coding regions resolved the evolutionary history of the Fucaceae, and allowed the dating of splitting events and tests of hypotheses concerning recent drivers of speciation. Diversification of the family could be placed in the Late-Mid Miocene. Four independent trans-Arctic colonisations were inferred, coincident with the split of the lineages leading to 1) *Pelvetia canaliculata*, 2) *Ascophyllum nodosum*, 3) the genus *Fucus*, and more recently 4) in the *F. distichus *species complex. Two dioecious lineages (originating the genera *Ascophyllum *and *Fucus*) evolved in the Atlantic/Arctic from hermaphroditic ancestors. Despite an earlier origin of the genus *Fucus*, most current species have evolved within a relatively short time frame starting 4-3 Ma in the Pleistocene. Both *Fucus *clades contain dioecious and hermaphroditic lineages, and recent speciation trends in clade 2 have given rise to hermaphroditic lineages from dioecious ancestors. Recent radiation in *Fucus *clade 2 coincides with divergence in physiological tolerance to environmental stresses and colonization of novel habitats at range edges, suggesting ecological speciation. In this clade, selfing lineages occur in the most extreme habitats, likely linked with reproductive assurance and the maintenance of local adaptation.

## Methods

### Taxa distribution and sampling

All species of *Silvetia *and the monotypic genera *Pelvetiopsis *and *Hesperophycus *occur exclusively in the Pacific. *Pelvetia *and *Ascophyllum *are monotypic genera occurring exclusively in the Atlantic. All species of *Fucus *occur in the Atlantic and its adjacent seas except *F. distichus *(*sensu lato*), which is also found in the Pacific. At least 3 individuals from each of the 6 genera of Fucaceae were used in all analyses except for *Ascophyllum *and *Pelvetia *(2 individuals; Additional file 2).

Samples were collected from several locations from where it was possible to transport specimens alive or deep frozen in dry ice to prevent RNA degradation: 1 Pacific, 7 North Atlantic, 1 Baltic and 1 Mediterranean regions were used (Additional file 2). Sequences from a previous study [11] were also added (shown in Additional file 2). Fresh material was lyophilized and samples were stored at room temperature with silica drying crystals prior to RNA extraction [56].

### RNA extraction, cDNA synthesis and amplification

Lyophilized tissue was powdered for 5 min on a Mixer Mill (MM 300 - Retsch, Germany) and total RNA was isolated using the extraction method as described in Pearson et al. [56]. RNA integrity was confirmed by electrophoresis on 1.2% denaturing agarose gels. For reverse transcription, a solution of 1 μg total RNA, 1 mM dNTPs and 5 μM oligo d(T) was denatured at 70°C for 5 min and placed on ice for > 1 min. First Strand Buffer, DTT (0.1 M), RNase OUT and *SuperScript™*

III (Invitrogen) were added, the mix was incubated at 55°C for 1-2 h, and the reaction was then heat-inactivated at 80°C for 10 min. A total of 13 coding regions were selected for sequence analysis (Additional file 1). Specific primers were designed from Expressed Sequence Tag (EST) consensus sequences in *F. vesiculosus *or *F. serratus *[57] using Primer3 software version 0.4.0 [58]. PCR was carried out in 20 μl reaction volumes containing 1-3 μl of cDNA (1/40 dilution) as template, 1.5 mM, 0.2 μM dNTPs, 0.5 μM of each primer and 1 U of Taq polymerase, with the following conditions: initial denaturation at 94°C for 3 min; 35 cycles of denaturation at 94°C for 20 s, annealing at 58°C for 90 s and a final extension at 65°C for 5 min. Products were sequenced at the Centre of Marine Sciences, University of Algarve (ABI 3130xl). The resulting chromatograms were analyzed using CodonCode Aligner v1.6.3 (CodonCode Corp., Dedham, Massachusetts, USA).

### Outgrouping procedure

The specificity of the cDNA primer sequences was too great to allow amplification of gene products outside the family Fucaceae, specifically for the sister families Xiphophoraceae and Hormosiraceae [23,38,59]. In order to include in the multi-gene phylogenetic estimations the sister families outside the Fucaceae, we used additional ITS sequence information from a previous study [23], but applyed more advanced methodological analyses. Those sequences were first re-aligned using MAFFT v6 [60], using the E-INS-i option recommended for sequences with multiple conserved domains and long gaps [61]. K80 plus I plus G was selected as the best model fit to the nucleotide data set based on AIC as implemented in MrModeltest [62]. ITS dataset was analysed using maximum likelihood and Bayesian approaches as described above (see multi-gene phylogenetic analyses section). This rooted phylogenetic resolution of the genera in the Fucaceae based on ITS sequences was used to infer the basal genera of the family Fucaceae. These genera, *Ascophyllum *and *Silvetia *were used as outgroup to root the multi-gene phylogenetic analyses aimed at inferring the order of the previously unresolved speciation events.

### Multi-gene phylogenetic analyses

The cDNA sequence dataset (Additional file 2) was aligned first using MAFFT v6 [60], using the G-INS-i option recommended for sequences with global homology [61]. Models of sequence evolution were selected based on Akaike Information Criterion (AIC) as implemented in MrModeltest v2.3 [62] for each of the 13 partitions defined by each gene: Hasegawa-Kishino-Yano model (HKY; [63]) was most appropriate for the 1st, 11th and 12th partitions, HKY plus I for 5th, 6th, 7th and 10th partitions and HKY plus G for 13th partition; Kimura 2-parameter (K80; [64]) for 8th and 9th partitions, plus I for 2nd partition; Symmetrical model plus G (SYM; [65]) for 3rd partition; and General Time Reversible (GTR; [66]) plus I for 4th partition. The combined data set was analyzed as one partition using the GTR model plus I and G.

Maximum likelihood bootstrap analysis with 999 replicates was performed to infer the phylogenetic relationships for the combined data set using PhyML v3.0.1 [67]. The substitution parameters were estimated over a neighbor-joining tree. Tree searching operations were set to best of nearest-neighbour interchange (NNI) with subtree pruning and regrafting (SPR). Partitioned Bremer support analysis [68] was performed using TreeRot v2 [69,70], in order to provide a measure of how the different partitions of the data contributed to the Decay index for each node in the context of the combined data analysis.

Bayesian inferences were performed with MrBayes v3.1.2 [71]. For the partitioned analysis, the substitution model and branch length estimates were allowed to vary independently in each partition. General forms of these models were used since there is a specific recommendation against the use of fixed priors for a and I in the software manual in order to explore more efficiently different values of these parameters. The number of generations was set to 10^6 ^with a sampling frequency of 1000 generations in a dual running process with four chains per run [72]. Majority rule consensus trees were computed after discarding the first 25% of the trees as burn-in, which were saved prior to MCMC convergence. Support for clades given by posterior probabilities was thus represented by the majority rule percentage.

### Evolutionary divergence time estimations

Two major problems preclude a well-defined fossil record for the brown algae: a) almost all brown algae are uncalcified; b) misidentification due to the morphological similarities with some members of the Rhodophyta [37]. Brown algae are known, however, from Miocene rocks in California and diatomaceous sediments in Central Europe [73,74]. Some of these can be directly compared to genera of the extant family Sargassaceae, as *Cystoseirites *(similar to *Cystoseira*) or *Paleohalidrys *(which has modern representatives) that are in the order Fucales, and provide a valuable framework for evolutionary parameter estimation and molecular dating of Fucaceae [37].

Likelihood ratio tests significantly rejected a strict (uniform) molecular clock for the alignment. Node age estimates were therefore obtained by Bayesian-calibrated phylogenies using an uncorrelated log-normal relaxed clock as suggested for protein-coding genes in a broad variety of species [75]. Gene-specific gamma-distributed rate heterogeneity among sites and partition into codon position allowed separate estimation of non-synonymous and synonymous sites [76]. The HKY model of evolution was defined as proposed by Shapiro et al. [77] for coding regions. Tree priors were fixed on the coalescent, using constant population size and expansion growth, and on Yule speciation models of demographic history. Monophyletic constraints were imposed for the nodes that were used to calibrate the evolutionary rates. Uniform priors were used for the tmrca of the Fucaceae family (Aquitanium to Tortonian age from Miocene epoch: minimum age of 7 Myr; maximum age of 23 Myr; based on [38] and previous analyses using 5.8S ribosomal nuclear DNA together with ITS-1 and ITS-2 regions; see Additional file 6). Tree priors were used for the tmrca of the Fucus genus. MCMC chains were run in BEAST v1.5.4 for 10^7 ^generations, with burn-in and sampling as described above [78]. Identical sequences or those with genetic distances less than 0.002 were removed prior to the analyses in order to prevent nodes without longitude on the dated reconstruction. Convergence and stationarity of the chains was evaluated by plotting trace files in Tracer v. 1.4 [78]. Phylogenetic trees were represented using R statistical software v2.13.0 [79] together with "ape v2.5-1" library [80].

### Estimation of ancestral character states and traits associated with lineage diversification

Methods to estimate the influence of species' traits on lineage diversification have improved with recent advances in the detection of phylogenetic signatures of state-dependent speciation and extinction [81]. In particular, hypotheses of trait acquisition for a binary character and asymmetry in the direction of trait evolution can now be tested through the formulation of a model [81]. For example, mating system is likely to confer unequal probabilities of speciation and extinction. Two states of the character were used for mating system evolution (dioecious *vs*. hermaphroditic), under one-parameter (MK1) and asymmetrical 2-parameter (MK2) Markov k-state models [82-84]. The binary state speciation and extinction model (BiSSE, [84]) was also used to avoid incorrect rejection of irreversible evolution [81].

Alternative hypotheses concerning geographic range evolution and diversification (Pacific *vs*. Atlantic), were also tested using a geographic state speciation and extinction model (GeoSSE; [85]). We applied the model to test the relative contributions of speciation, extinction, and dispersal to diversity differences between oceans [85]. We also considered different combinations of state-independent and state-dependent diversification, and dispersal (Table 2).

BiSSE and GeoSSE model assumptions were satisfied through the use of the best rooted tree based on the dated ITS and multi-gene phylogenies: i) rooted phylogenetic tree with branch lengths; ii) contemporaneous terminal taxa and; iii) ultrametric tree [81]. Characters were binary with known state for each of the terminal taxa. Models were fitted by maximum likelihood nonlinear optimization from a heuristic starting point based on the character-independent birth-death model. Model results were evaluated and compared using the logarithm of the likelihood and the AIC values for the final fitted models. Ancestral character states and the associated uncertainty were also estimated from the scaled likelihood of each character state. Analyses were carried out using the R statistical software [79], with "diversitree v0.7-2" and "ape v2.5-1" packages [80,85-87].

The dispersal-extinction-cladogenesis (DEC) likelihood model was also implemented to infer geographic ancestry and estimate rates of dispersal and local extinction [88,89]. Unconstrained and stratified biogeographical models were considered. The latter model stratified the phylogeny into different time slices, reflecting the Bering Strait configuration over time while considering divisions that retained enough phylogenetic events [90]. Five time slices were chosen that reflect the hypothesized openings of the Bering Strait during the history of Fucaceae: between 13 and 11 Ma, between 7.3 and 6.6 Ma, between 5.5 Ma and 4.0, between 3.6 Ma and 3.2, and between 2.5 and the present day (see Figure 2 for a detailed time-placement of the recurrent opening events [18,20]). For each time slice, we defined a Q matrix in which transition rates were made dependent on the geographical connectivity between areas (i.e. opening and closing of the Bering Strait). Lagrange analyses were configured using the web application from the same authors (URL: http://www.reelab.net/lagrange/configurator;[88,89]) and run locally using Lagrange v.20110117 [89]. Results were summarized and plotted using the R statistical software [79] with the "ape v2.5-1" package [80].

## Abbreviations

AIC: Akaike information criteria; BiSSE: Binary state speciation and extinction model; DEC: Dispersal-extinction-cladogenesis model; GeoSSE: Geographic state speciation and extinction model; ITS: Internal Transcribed Spacer; Myr: Million years; Ma: Million years ago; mrca: Most recent common ancestor; SNP: Single nucleotide polymorphism; v: Software version; Kyr: Thousands years; tmrca: Time to the most recent common ancestor.

## Authors' contributions

The study was conceived by GAP and EAS. GAP supervised the project. CFM performed all the laboratory work. FC performed all the phylogenetic analyses. FC, GAP and EAS wrote the manuscript. All authors read and approved the final manuscript.

## Supplementary Material

Additional file 1 **Incorporated cDNA sequences**. Annotations of coding region transcripts used in this study. Total and used length expressed in base pairs (bp) and amino acids (aa) as well as primer sequences are presented. As P, we indicate the partition number for each region used in mixed analyses. Click here for file

Additional file 2 **Sampling sites and GeneBank accession numbers of all incorporated cDNA sequences**. Click here for file

Additional file 3 **Separate analyses of ribosomal nuclear DNA together with ITS regions (see Methods section: estimation of ancestral character states)**. Phylogenetic reconstructions using 5.8 S ribosomal nuclear DNA together with ITS-1 and ITS-2 regions (re-analysis of data from [23] after testing for best fit model). Values shown are the 50% majority rule percentage of support for clades given by Bayesian posterior probabilities from one million generation MCMC analysis (above) and the 50% majority rule consensus tree of maximum likelihood bootstraps (below). *H. banskii *was used as outgroup to root the phylogenetic reconstructions (as in [23]). These results were also used to root the multi-gene phylogenetic trees since the specificity of primers used to amplify transcriptomic regions in the Fucaceae did not allow amplification outside this group. Topology is based on maximum likelihood reconstruction. Click here for file

Additional file 4 **Bayesian dating of Fucaceae diversification using cDNA**. Full tree showing the Bayesian dated phyloreconstruction using the 13 coding loci. Node ages in million years (Myr) correspond to the time scale at the bottom of the figure. Click here for file

Additional file 5 **Multi-gene phylogenetic reconstruction from cDNA (see Methods section: Multi-gene phylogenetic analyses of cDNA sequences) including introgressed sequences**. Multi-gene phylogenetic relationships as shown in Figure 1 but adding sequences of *F. guiryi *from northern Portugal, where the species co-occurs in sympatry with *F. vesiculosus *and *F. spiralis*, creating an introgressed range for *F. guiryi *that continues northwards [11,25,26,47]. Methods are the same as described for Figure 1. Comparison of this tree with Figure 1 illustrates the effect of introgressed contact regions in preventing phylogenetic resolution, by confounding vertical lineage splitting with horizontal introgressive mixing. Click here for file

Additional file 6 **Bayesian dating of Fucaceae diversification using nuclear ribosomal DNA: the 5.8 S gene together with ITS-1 and ITS-2 regions**. Bayesian dated phyloreconstruction using nuclear ribosomal DNA, the 5.8 S together with ITS-1 and ITS-2 regions. Node ages in million years (Myr) with their 95% HPD interval correspond to the time scale at the bottom of the figure. Node age estimates were obtained using an uncorrelated log-normal relaxed clock under GTR model of evolution. Tree priors were fixed on Yule speciation model of demographic history. One individual of *Cystoseira neglecta, C. osmundacea *and *C. setchellii *species were included as representatives of the family Sargassaceae for the inferences ([94]; accession numbers: AY542816, AY542819 and AY542812). Monophyletic constraints were imposed for the nodes that were used to calibrate the evolutionary rates. Normal priors were used for the times to the most recent common ancestor (tmrca) of Fucaceae and Sargassaceae families (Medium Chattium to Aquitanium age from Miocene epoch: mean 22.5 million years (Myr); standard deviation 2.5 Myr [37]). Results were processed as described in the methods section. Click here for file
