# Supplementary material for: Driving south: a multi-gene phylogeny of the brown algal family Fucaceae reveals relationships and recent drivers of a marine radiation
Source: BMC Evol Biol. 2011 Dec 21;11:371. doi: 10.1186/1471-2148-11-371 (PMC3292578; doi:10.1186/1471-2148-11-371)
Supplement: Additional file 1 — Incorporated cDNA sequences. Annotations of coding region transcripts used in this study. Total and used length expressed in base pairs (bp) and amino acids (aa) as well as primer sequences are presented. As P, we indicate the partition number for each region used in mixed analyses. [file 1471-2148-11-371-S1.DOC]

| **P** | **Gene name** | **Locus name** | **Description** | **length bp** | **length aa** | **used length** | **Primers (5'-3')** |
| --- | --- | --- | --- | --- | --- | --- | --- |
| 1 | BiP1 | HS552 | 78 kDa glucose-regulated protein homologue | 303 | 101 | 186 | F CCTGTGTGCGTCTCGCCTCTTTTC  R GTCAAGGACCGCATCGAC |
| 2 | clpB | - | casein lytic proteinase | 669 | 223 | 507 | F GCGTCAGTAGCTTCCAAAGT  R CTGCCTACATGTTCGACACC |
| 3 | clpC | HS5978 | ATP-dependent Clp protease ATP-binding subunit clpA | 639 | 213 | 600 | F CTCCACCGAAGCCAATTCCTTGTG  R CGTGCTCAAATGGGTGCTA |
| 4 | clpP | - | ATP-dependent clp protease, proteolytic subunit clpP | 462 | 154 | 399 | F TGCGAACTGAGAAATGTGCGATGTC  R AAACAATGACCCGGAGAAAG |
| 5 | eIF3l | HS700 | eukaryotic translation initiation factor 3, subunit 6 interacting protein | 573 | 191 | 564 | F GTGTCCACGACGAGCCCATAAACG  R AAACGGCGGACAAGATGAT |
| 6 | HSP90_1 | HS597 | Heat shock protein 90 family | 303 | 101 | 309 | F CTACGCGCCGTCTCGAAAACAGAC  R CTCAGGACAGCGGAGCAT |
| 7 | HSP90_2 | HS870 | Heat shock protein 90 family | 513 | 171 | 456 | F CGACCACCTCTGCATCCTTCACC  R CAAGCCATCGGAGAGTACAAG |
| 8 | mpv17l2 | HS544 | Mpv17-like protein | 327 | 109 | 321 | F AAACTACGGTTGCCGCACTCAACG  R CGATCAGACCATCTGGAACC |
| 9 | TTC1 | 6A15 | Tetratricopeptide repeat protein 1 | 408 | 136 | 330 | F CAAGCACCACTGGAACGACGATAG  R GACGGAGAGGAACACAACAAAG |
| 10 | STI1 | HS718 | Hsp70/Hsp90 organizing protein homolog | 669 | 223 | 459 | F GGGCCTTTTCACACGCTGCCTTA  R AAAGACGAGAAGGACGACGA |
| 11 | CCT4 | TCP1delta | T-complex protein 1, delta subunit | 231 | 77 | 183 | F CCTTCCAAGCAGGACAGCACCA  R TACACCCTCGCGGAGAAC |
| 12 | CCT-epsilon | TCP1epsilon | T-complex protein 1, epsilon subunit | 432 | 144 | 354 | F AAAGCCCCTCGCGATGTTTATCGT  R GAGGCGAAACGCTCTCTC |
| 13 | PXMP2/4_1 | 1E12 | PXMP2/4 family protein 2 | 540 | 180 | 210 | F AAAGAAGCCGCCGCAGTTGGTGTA  R GGTACACCGCTTTGCTAGAGT |
