# Supplementary figures and images for: Driving south: a multi-gene phylogeny of the brown algal family Fucaceae reveals relationships and recent drivers of a marine radiation
Source: BMC Evol Biol. 2011 Dec 21;11:371. doi: 10.1186/1471-2148-11-371 (PMC3292578; doi:10.1186/1471-2148-11-371)

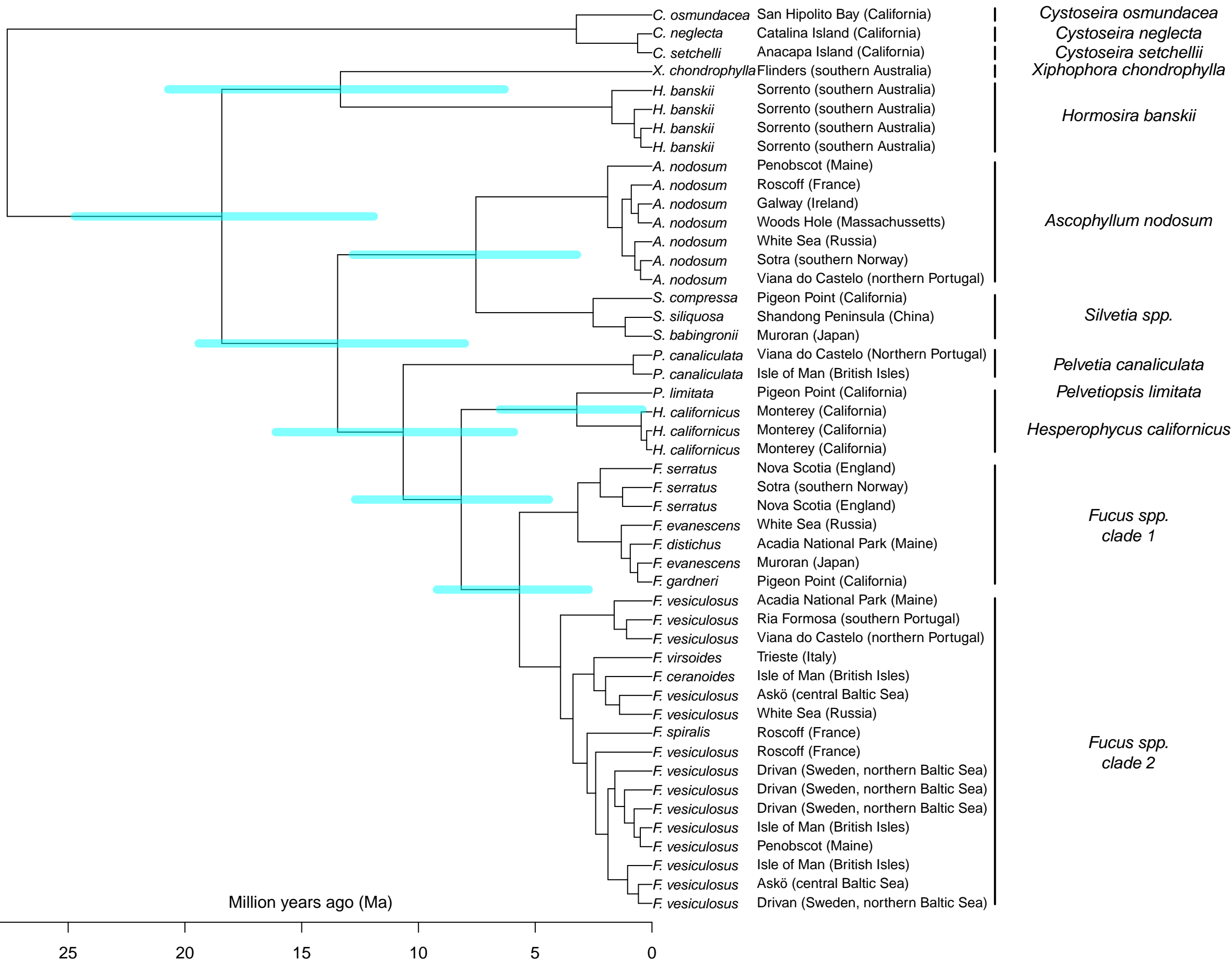

Supplement: Additional file 6 — Bayesian dating of Fucaceae diversification using nuclear ribosomal DNA: the 5.8 S gene together with ITS-1 and ITS-2 regions. Bayesian dated phyloreconstruction using nuclear ribosomal DNA, the 5.8 S together with ITS-1 and ITS-2 regions. Node ages in million years (Myr) with their 95% HPD interval correspond to the time scale at the bottom of the figure. Node age estimates were obtained using an uncorrelated log-normal relaxed clock under GTR model of evolution. Tree priors were fixed on Yule speciation model of demographic history. One individual of Cystoseira neglecta, C. osmundacea and C. setchellii species were included as representatives of the family Sargassaceae for the inferences ([94]; accession numbers: AY542816, AY542819 and AY542812). Monophyletic constraints were imposed for the nodes that were used to calibrate the evolutionary rates. Normal priors were used for the times to the most recent common ancestor (tmrca) of Fucaceae and Sargassaceae families (Medium Chattium to Aquitanium age from Miocene epoch: mean 22.5 million years (Myr); standard deviation 2.5 Myr [37]). Results were processed as described in the methods section. [file 1471-2148-11-371-S6.PDF]
